# Residual Expression of the Reprogramming Factors Prevents Differentiation of iPSC Generated from Human Fibroblasts and Cord Blood CD34+ Progenitors

**DOI:** 10.1371/journal.pone.0035824

**Published:** 2012-04-24

**Authors:** Verónica Ramos-Mejía, Rosa Montes, Clara Bueno, Verónica Ayllón, Pedro J. Real, René Rodríguez, Pablo Menendez

**Affiliations:** Centre Pfizer-Universidad de Granada-Junta de Andalucía for Genomics, Oncological Research (GENyO), Granada, Spain; Hemocentro de Ribeirão Preto, HC-FMRP-USP, Brazil

## Abstract

Human induced pluripotent stem cells (hiPSC) have been generated from different tissues, with the age of the donor, tissue source and specific cell type influencing the reprogramming process. Reprogramming hematopoietic progenitors to hiPSC may provide a very useful cellular system for modelling blood diseases. We report the generation and complete characterization of hiPSCs from human neonatal fibroblasts and cord blood (CB)-derived CD34+ hematopoietic progenitors using a single polycistronic lentiviral vector containing an excisable cassette encoding the four reprogramming factors Oct4, Klf4, Sox2 and c-myc (OKSM). The ectopic expression of OKSM was fully silenced upon reprogramming in some hiPSC clones and was not reactivated upon differentiation, whereas other hiPSC clones failed to silence the transgene expression, independently of the cell type/tissue origin. When hiPSC were induced to differentiate towards hematopoietic and neural lineages those hiPSC which had silenced OKSM ectopic expression displayed good hematopoietic and early neuroectoderm differentiation potential. In contrast, those hiPSC which failed to switch off OKSM expression were unable to differentiate towards either lineage, suggesting that the residual expression of the reprogramming factors functions as a developmental brake impairing hiPSC differentiation. Successful adenovirus-based Cre-mediated excision of the provirus OKSM cassette in CB-derived CD34+ hiPSC with residual transgene expression resulted in transgene-free hiPSC clones with significantly improved differentiation capacity. Overall, our findings confirm that residual expression of reprogramming factors impairs hiPSC differentiation.

## Introduction

Human fibroblasts were the first cell type successfully reprogrammed by ectopic expression of OCT4, SOX2, KLF4 and c-MYC (OKSM) [1], [2], [3], [4], [5], [6]. Subsequently, human induced pluripotent stem cells (iPSC) have been derived from different tissues, and further studies have shown that the age, origin and cell type used as target for reprogramming affects the reprogramming efficiency, eventually requiring the expression of fewer factor [7], [8], [9]. Cord blood (CB) represents a source enriched with hematopoietic, mesenchymal and endothelial precursors. Access to CB units is very feasible by harnessing those CB units which do not reach the minimum quality criteria to be frozen and stored for potential future use in CB allogeneic transplantation and would otherwise be discarded at the public CB banks. Besides, CB cells are young newborn cells expected to accumulate minimal somatic mutations as compared to other somatic tissues sourced from older subjects, and they display the immunological immaturity of neonatal cells [10].

Recently, Haase *et al* [11] derived hiPSC from CB-derived endothelial cells while Giorgetti *et al* [7] also succeeded in the derivation of hiPSC from a CB-derived CD133+ cell subset which contains hematopoietic progenitor cells committed to myeloid but not lymphoid lineages [12], [13], [14], [15]. Furthermore, Takenaka *et al* [16] very recently derived hiPSC from CB-derived CD34+ cells upon retroviral over-expression of OKSM factors and showed that further p53 depletion robustly enhanced reprogramming efficiency. Finally, Ye *et al* [17] also derived hiPSC from blood cells and CB-derived CD34+ cells using four independent retroviral vectors: pMXs-Oct4, pMXs-Sox2, pMXs-Klf4 and pMXs-c-myc.

Despite these advances, to the best of our knowledge, hiPSC have not been generated yet from CB-derived primitive CD34+ cells using a single polycistronic lentiviral vector based on 2A and IRES sequences expressing the four reprogramming factors (OKSM) under a constitutive promoter [18], [19], thus minimizing the number of proviral insertions. In addition, even though important information is available in the mouse [20], [21], it has not been robustly assayed in humans whether the cellular origin influences the *in vitro* differentiation potential of hiPSC lines. That is, whether those hiPSC derived from a certain somatic cell type are more prone to differentiate towards the same specific lineage from which they were initially derived, or on the contrary, hiPSC lines derived from different cell types differentiate equally towards certain lineage, regardless of the origin of the reprogrammed somatic cell.

Here, we describe the successful derivation of hiPSC from CB-CD34+ cells using a single polycistronic lentiviral vector expressing OKSM based on 2A and IRES sequences [18], [19]. Interestingly, the ectopic expression of OKSM was fully silenced upon reprogramming in some hiPSC clones while other hiPSC clones failed to silence the transgenes expression. The inability/ability of hiPSC to silence the reprogramming factors was not associated to the cell type/tissue origin. However, continued transgene expression seriously impaired hematopoietic and early neuroectoderm differentiation potential, indicating that residual expression of the reprogramming factors compromises hiPSC differentiation. This was confirmed in transgene-free hiPSC clones generated from CB CD34+ hiPSC with residual transgene expression by Cre-mediated excision of the provirus cassette, which resulted in significantly improved differentiation capacity.

## Materials and Methods

### CB collection and CD34+ cell isolation

Umbilical CB samples from healthy newborns were obtained from the Andalusian Public Cord Blood Bank upon approval by our local (University of Granada) Ethics and Biozahard Board Committee (ABR/JFJ/S-23). All human samples were obtained upon informed consent given by the parents. Mononuclear cells were isolated using Ficoll-Hypaque (GE Healthcare, Stockholm, Sweden). After lysing the red blood cells (Lysis solution, StemCell Technologies, Vancouver, Canada), CD34^+^ cells were purified by magnetic bead separation using the human CD34 MicroBead kit (Miltenyi, Munich, Germany) and the AutoMACS Pro separator (Miltenyi) as per manufacturer's instructions [22], [23]. Post sorting purity was higher than 95% (data not shown). After washing in phosphate-buffered saline (PBS), CD34^+^ cells were plated in liquid culture: Stem Span medium (Stem Cell Technologies) supplemented with SCF (100 ng/mL), FLT3L (100 ng/mL) and IL-3 (10 ng/mL) (Peprotech, London, UK) [23], [24]. Furthermore, early passage (p5–p10) human embryonic fibroblasts (HEFs) obtained from ATCC (IMR90) were used and grown in DMEM+10% FBS [25].

### Vectors, lentiviral production and transduction of fibroblasts and CB-derived CD34+ hematopoietic stem/progenitor cells (HSPC)

**Figure 1:**
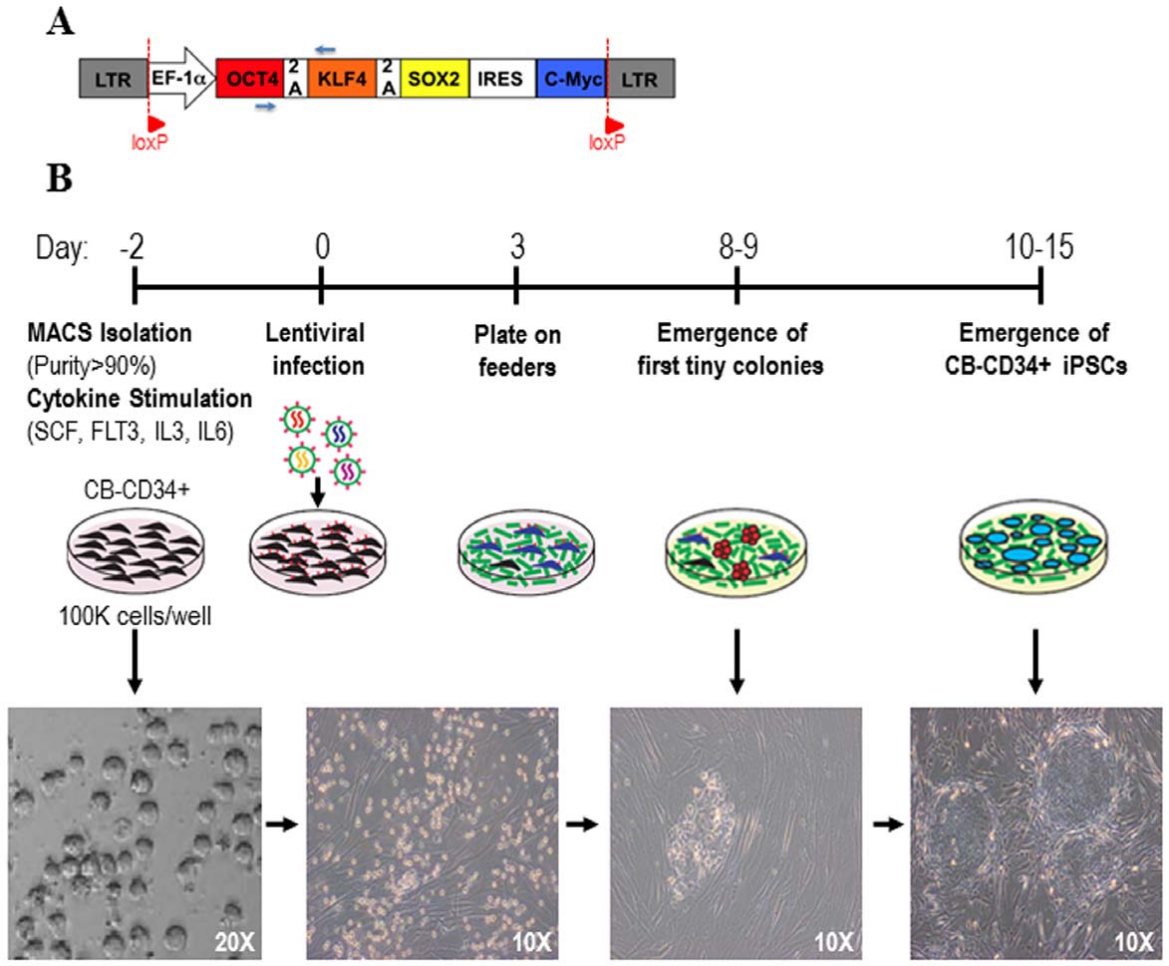
Lentiviral vector design and experimental strategy used in the generation of hiPSC from CB-derived CD34+ HSPCs. (**A**) Structure of the policistronic lentivector used for iPSC generation. EF1α is the constitutive promoter. The four reprogramming factors (Oct4, Klf4, Sox2, c-myc; OKSM) are expressed as independent transcripts through the 2A and IRES sequences. The small blue arrows identify the primers used to determine the silencing of the ectopic expression of the reprogramming factors. (**B**) Timeline of fibroblast and CB-CD34+ reprogramming. CD34+ cells were MACS-isolated and pre-stimulated with hematopoietic cytokines. Two days later, CB-CD34+ cells were infected. After 3 days, infected cells were transferred onto feeders. Small adherent colonies were observed around day 8–9. hESC-like colonies were identifiable after 10–15 days.

CB-derived CD34+ HSPCs (1×10^5^ cells/mL) were pre-stimulated for 48 h in Stem Span medium supplemented with SCF (100 ng/mL), FLT3L (100 ng/mL) and IL-3 (10 ng/mL) in fibronectin-coated plates (Becton Dickinson, San José, CA). hiPSC were generated by infection of either pre-stimulated CD34+ HSPCs or fibroblasts with high-titer (MOI = 20–30) policistronic lentiviral vectors co-expressing the four human reprogramming factors Oct4, Klf4, Sox2 and c-myc (OKSM) under the constitutive promoter (EF1α) using 2A and IRES elements as shown in **Figure 1A**. Viral particles pseudotyped with the VSV-G protein were generated on 293T cells (purchased from the ATCC; www.atcc.org) using a standard calcium-phosphate transfection protocol and were concentrated by ultracentrifugation [24], [26], [27]. Cells were infected overnight and the following day the viral supernatant was removed and transduced fibroblast or CD34+ HSPCs were washed and allowed to expand for 3 days before transferring them onto irradiated (4000 cGy) feeders (MEFs). Once transferred onto feeders, the cultures were maintained in KO-DMEM supplemented with 20% serum replacement, 1% nonessential amino acids, 1 mM L-glutamine, and 0.1 mM β-mercaptoethanol (all from Invitrogen) and 50 ng/mL of basic fibroblast growth factor (bFGF) (Miltenyi). Identification of the first emerging hiPSC colonies (10–15 days after viral infection) and passage onto fresh feeders was done as previously described (**Figure 1B**) [6], [25]. Approval from the Spanish National Pluripotent Ethical Committee was obtained to work with hiPSC (ABR/JFJ/S-23).

### Total RNA purification and Reverse-Transcriptase-Polimerase Chain Reaction (RT-PCR)

Total RNA extraction and RT-PCR reactions were done as previously described [28]. Briefly, total RNA was extracted from hiPSC or differentiating embryoid bodies (hEBs) using Total RNA Purification Kit (Norgen, Canada). First-strand cDNA synthesis was performed using the First-Strand cDNA Synthesis Kit (Amersham) and the expression of pluripotency-associated transcription factors (Oct-4, Nanog, Rex-1 and Sox2) and early mesoderm and ectoderm factors (Brachyury and Pax6, respectively) was assessed by end-point RT-PCR as described [28]. GAPDH was used as a housekeeping gene. The following PCR conditions were used: 5 min at 94°C, 35 cycles of 30 seconds at 94°C followed by 50 seconds at 60°C and 50 seconds at 72°C and a final extension of 10 min at 72°C. The PCR products were resolved in 1% agarose gel. Primer sequences were as follows: Oct3/4: 5′-TCTGCAGAAAGAACTCGAGCAA-3′, and 5′-AGATGGTCGTTTGGCTGAACAC-3′; Nanog: 5′-TGCAGTTCCAGCCAAATTCTC-3′, and 5′-CCTAGTGGTCTGCTGTATTACATTAAGG-3′; Sox-2: 5′-CCCCCGGCGGCAATAGCA-3′, and 5′-TCGGCGCCGGGGAGATACAT-3′; Rex-1: 5′-CAGATCCTAAACAGCTCGCAGAAT-3′, and 5′-GCGTACGCAAATTAAAGTCCAGA-3′; Brachyury: 5′-ATGAGCCTCGAATCCACATAGT-3′ and 5′-TCCTCGTTCTGATAAGCAGTCA-3′; Pax6: 5-CCGGCAGAAGATTGTAGAGC-3 and 5-CGTTGGACACGTTTTGATTG-3 and GAPDH: 5′-GAAGGTGAAGGTCGGAGTC-3′, and 5′-GAAGATGGTGATGGGATTTC-3′.

In order to detect the expression/silencing of the reprogramming factors encoded from the lentiviral vector, the following primers were used: Forward (on Oct-4): 5′-GCCTTTCCCCCTGTCTCCGTC-3′ and Reverse (on Klf-4) 5′-GGCTCCGCCGCTCTCCAGGTCTG-3′. The PCR conditions were as follows: 2 min at 95°C, 40 cycles of 30 s at 94°C followed by 30 s at 60°C and 60 s at 72°C, and a final extension of 5 min at 72°C.

### Embryoid body (EB) formation and *in vitro* differentiation

Human iPSC were harvested, transferred to low-attachment plates and allowed to differentiate spontaneously through EB formation in KO-DMEM supplemented with 20% FBS (Invitrogen), 1% L-glutamine, 0.1 mM non-essential amino acids and 0.1 mM β-mercaptoethanol but without bFGF with media changes every 4 days. For spontaneous differentiation, day 21 EBs were spun down, fixed with 4% paraformaldehyde for 10 minutes and embedded in paraffin and sections were stained with hematoxylin and eosin (H&E) [29], [30], [31]. For each staining, three sections per specimen were taken. Immunostaining for lineage-specific markers was performed as described below.

### Teratoma formation

Animal protocols were approved by the Granada University Hospital Council On Animal Care and Experimentation (CEEA-2011-361). *In vivo* pluripotency was tested as previously described [31]. In brief, hiPSC lines were harvested with Collagenase IV. hiPSC were implanted beneath the testicular capsule of a young (6–8 weeks) NOD/SCID IL2Rγ^−/−^ male mouse (The Jackson Lab, Bar Harbor, MA). Teratoma growth was determined by palpation every week, and the mice were sacrificed 7–10 weeks after implantation. Teratomas were fixed, embedded in paraffin and sections were stained with H&E. Immunostainings for germ-layer specific markers were performed as described below.

### Immunocytochemistry

EB and teratoma sections were incubated (1 hour at room temperature) with primary antibodies for anti-βIII-tubulin (DAKO; 1∶100), pan-cytokeratin (DAKO; 1∶100) and anti-smooth muscle actin (Chemicon; 1∶100). Then, the sections were incubated with a biotinylated secondary antibody (30 minutes at room temperature) and a streptavidin peroxidase complex (30 minutes at room temperature) (Vector Laboratories Inc). The immunostaining was visualized using diaminobenzidine and counterstained with H&E. The washing steps were done in PBS. Negative controls were prepared by replacing the primary antibody by PBS.

### Flow Cytometry

Expression of pluripotency-associated markers in established hiPSC was determined by flow cytometry using antibodies against SSEA-3, SSEA-4 (Developmental Studies Hybridoma Bank, University of Iowa, USA), TRA-1-60 and TRA-1-81 (Chemicon, CA) and Oct-4 (Pharmigen). An irrelevant isotype-match antibody was always used. Tripsin-dissociated hiPSC lines were suspended in PBS+3%FBS at a concentration of 2–5×10^4^ cells per 100 µL and incubated with the specific primary antibody for 30 minutes at 4°C. After washing with PBS+3%FBS, cells were incubated with 2.5 µL of FITC- or PE-conjugated goat anti-mouse IgG antibody (Immunotech, Marseille, France). After 15 minutes at RT, the cells were washed and stained with 7-aminoactinomycin D (7-AAD) (Immunotech) for 5 minutes at RT. Live cells identified by 7-AAD exclusion were analyzed for expression of SSEA-3, SSEA-4, Tra-1-60, Tra-1-81 and Oct-4 using a FACSCanto-II flow cytometer equipped with FACSDiva software (Becton Dickinson) [32], [33].

### G-Banding

Karyotype analysis was performed according to previous standardized protocols [34], [35], [36]. At least 20 metaphases of each sample were counted.

### Cre-mediated excision of the reprogramming factors in hiPSC

Transgene-free hiPSC were generated by excision of the LoxP-flanked sequences by infection of hiPSC with adenoviral vectors expressing the Cre-recombinase gene under the control of the CMV promoter (Ad-CMV-Cre), as previously described [37]. The successful gene knockdown and generation of transgene-free hiPSC after the excision of the LoxP regions was confirmed by genomic PCR. For genomic PCR, total DNA was extracted from hiPSC and hEBs using the DNAeasy kit (Qiagen, Alameda, CA). 200 ng of DNA were used for each PCR reaction. The PCR conditions were as follows: 2 min at 95°C, 40 cycles of 30 s at 94°C followed by 30 s at 60°C and 60 s at 72°C, and a final extension of 5 min at 72°C. The following primers were used: Forward: 5′-GCCTTTCCCCCTGTCTCCGTC-3′ and Reverse 5′-GGCTCCGCCGCTCTCCAGGTCTG-3′.

### hiPSC differentiation towards hematopoietic lineage

At confluence, hiPSC maintained feeder-free were treated with collagenase IV and scraped off of the Matrigel attachments using a pipette. They were then transferred to low-attachment plates (Corning, NY) to allow hEB formation (iPSC colonies cannot attach any longer in low-attachment plates and form this differentiating structures termed EBs) by overnight incubation in differentiation medium consisting of KO-DMEM supplemented with 20% non-heat-inactivated FBS, 1% nonessential amino acids, 1 mM L-glutamine, and 0.1 mM β-mercaptoethanol and without bFGF. The medium was changed the next day (day 1) with the same differentiation medium supplemented with hematopoietic cytokines [300 ng/mL SCF, 300 ng/mL Flt-3 ligand, 10 ng/mL IL-3, 10 ng/mL IL-6, and 50 ng/mL G-CSF and 25 ng/mL BMP-4] [22], [38], [39], [40]. EBs were dissociated using collagenase B (Roche Diagnostic, ON, Canada) for 2 hours at 37°C followed by 10 minutes incubation at 37°C with enzyme-free Cell Dissociation Buffer (Invitrogen) at day 15 and 22 of development. Single cell suspension was obtained by gentle pipetting and passage through a 70-µm cell strainer (Becton Dickinson) and the dissociated cells were stained with anti-CD34-FITC and anti-CD45-APC (Miltenyi) antibodies and 7AAD. Live cells identified by 7-AAD exclusion were analyzed using a FACSCanto II flow cytometer equipped with FACSDiva software (Becton Dickinson). Primitive and total blood cells were identified as CD45+CD34+ and CD45+CD34−, respectively [41], [42], [43], [44].

### Colony Forming Unit (CFU) formation

Human clonogenic progenitor assays were performed by plating 1000 cells from day 15 hEBs into methylcellulose H4230 (Stem Cell Technologies) supplemented with recombinant human growth factors: 50 ng/mL SCF, 3 units/mL erythropoietin, 10 ng/mL GM-CSF, and 10 ng/mL IL-3. Cells were incubated at 37°C in a 5% CO_2_ humidified atmosphere and colonies counted at day 14 of CFU assay using standard morphological criteria [45], [46]. CFUs were harvested from the methylcellulose, dissociated in cold PBS, washed in PBS and stained with anti-CD15-FITC, anti-CD33-PE and anti-CD45-APC (Becton Dickinson) for flow cytometry phenotyping

### Early neural differentiation

For neural differentiation, the protocol was slightly modified from that described by Pankratz et al [47]. Briefly, hiPSC lines were grown in suspension as hEBs in growth medium for 4 days. The hEBs were then cultured in neural medium composed by Dulbecco's modified Eagle's medium/F12, nonessential amino acids, 2 µg/mL heparin, and the neural cell supplement N2 (Gibco, Billings, MT) for 3 additional days. Early neural differentiation was evaluated at day 8 of culture by dissociating and staining hEBs single cells with the neural embryonic antigen A2B5 using a mouse anti-human A2B5 (1∶25 dilution; Miltenyi) or the corresponding isotype control. The secondary antibody was a FITC-conjugated goat anti-mouse antibody.

## Results

### Reprogramming CB-CD34+ HSPCs with OKSM factors using a single policistronic lentivector containing 2A and IRES sequences

The majority of HSPCs are quiescent under physiological conditions [48]. Because integration and expression of retroviral constructs requires mitotic division of target cells, we aimed at generating hiPSC using lentiviral vectors which are capable of infecting both quiescent and cycling cells [48]. In order to reprogram CB-CD34+ HSPCs to pluripotency using a single policistronic lentiviral vector driving the expression of OKSM under the constitutive EF1α promoter (**Figure 1A**), 1×10^5^ cytokine pre-stimulated CD34+ HSPCs were infected overnight with high-titer concentrated viral supernatant. After 16 hours, the virus-containing media was washed away and the infected cells were allowed to expand and recover for two further days. Three days after infection, CB-CD34+ cells were transferred onto irradiated feeders and cultured in hESC typical culture medium (**Figure 1B**). As early as 8–9 days after postinfection tiny colonies started to appear in CD34+ cultures transduced with OKSM lentiviruses (**Figure 1B**). At 10–15 days post-infection, we found that some of these initial small colonies were transient and they disappeared, likely indicating that they constituted partially reprogrammed iPSC (pseudo-iPSC) unable to survive and expand over passages [49], [50]. However, other colonies expanded and exhibited typical hESC morphology with sharp borders, and were comprised of small, tightly packed cells with large nuclei (**Figure 1B**). Overall, 21 small colonies (pseudo-iPSC) were obtained from 1×10^5^ infected CD34+ HSPCs (transient reprogramming efficiency of 1∶4761 CD34+ cells; 0.02%). Nevertheless, the reprogramming efficiency, as judged by the number of fully reprogrammed iPSC was of 1 iPSC: 50.000 CD34+ cells, resulting in a 0.0002% reprogramming efficiency.

### *In vitro* and *in vivo* characterization of the fibroblast and CB-CD34+ hiPSC lines

**Figure 2:**
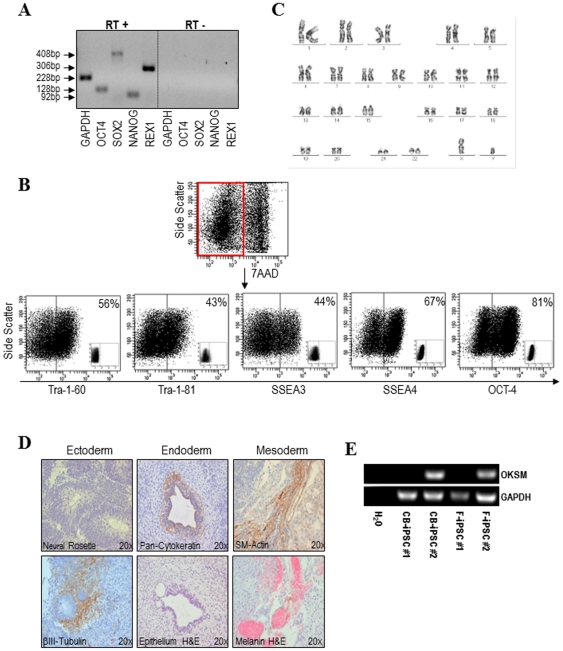
Characterization of hiPSC generated either from fibroblasts or CB-CD34+ HSPCs. All the hiPSC lines generated from either fibroblasts or CB-CD34+ HSPCs express the pluripotency markers Oct4, Sox2, Nanog and Rex1 (**A**), the pluripotent surface markers Tra-1-60, Tra-1-81, SSEA-3 and SSEA-4, as well as nuclear Oct4 (**B**) and display an euploid karyotype (**C**). All hiPSC lines differentiated *in vivo* (teratoma formation) (**D**) into tissues representing the three germ layers: ecto-, meso- and endoderm. Immunocytochemistry pictures are shown at 20× magnification. (**E**) RT-PCR confirming that CB-iPSC#2 and F-iPSC#2 did not silence OKSM factors after >20 passages.

Two hiPSC clones were generated from fibroblasts (termed F-iPSC #1 and F-iPSC #2) [25] and other two from CB-CD34+ HSPCs (termed CB-iPSC#1 and CB-iPSC#2). All hiPSC have been equally cultured for over 20 passages in feeder-free conditions using matrigel and conditioned media and have successfully survived repetitive freeze-thaw processes (data not shown). In addition, all hiPSC lines expressed the ESC-associated transcription factors Oct-4, Nanog, Rex-1 and Sox-2 (**Figure 2A**), and the pluripotency-associated surface markers Tra-1-60, Tra-1-81, SSEA-3, SSEA-4 and nuclear Oct-4 (**Figure 2B**). Importantly, after 20 passages, they all remained karyotypically euploid (**Figure 2C**).

Functionally, these hiPSC lines successfully differentiated *in vitro* through EB formation into tissues representing the three germ layers: ectoderm (neuroepithelium), mesoderm (smooth muscle-α-actin+ cells) and endoderm (pan-cytokeratin+ cells) (**Figure S1**). *In vivo*, all hiPSC lines formed teratomas ∼6 to 8 weeks after inoculation [31]. These complex and disorganized tumours contained a variety of tissues representing the three germ layers (**Figure 2D**), demonstrating the pluripotent features of these hiPSC lines.

We next wanted to verify proper proviral silencing upon reprogramming. Unexpectedly, we found that two hiPSC lines (CB-iPSC#1 and F-iPSC#1) fully silenced the reprogramming transgenes whereas the other two hiPSC lines (CB-iPSC#2 and F-iPSC#2) failed to silence the transgene expression over a period of 20 passages (**Figure 2E**). Of note, the inability of the different hiPSC to silence the reprogramming factors was not associated to the cell type/tissue origin (fibroblasts *versus* CB-CD34+ HSPCs).

### Residual transgene expression prevents hematopoietic and neuroectoderm differentiation of hiPSC

**Figure 3:**
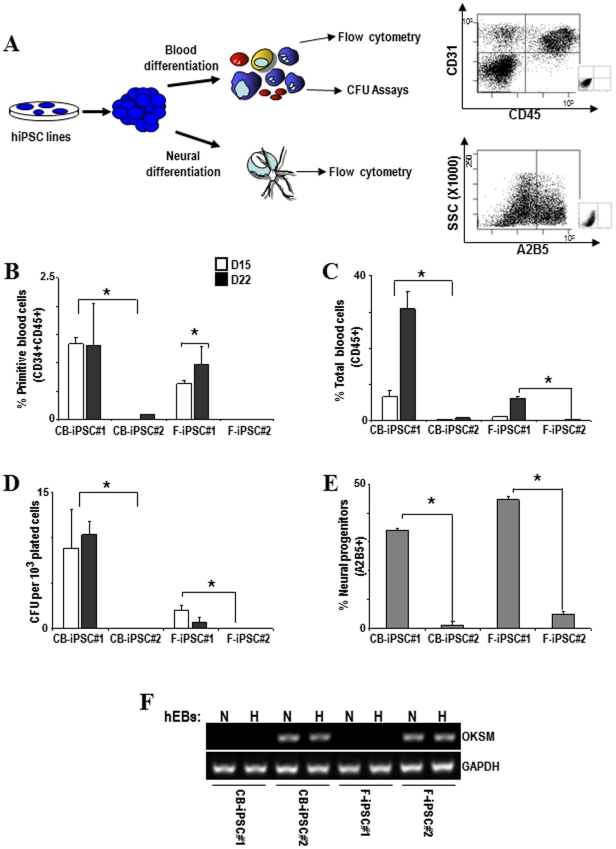
Residual expression of ectopic reprogramming factors prevents differentiation of hiPSC lines. (**A**) Schematic of the hematopoietic and neural differentiation protocol from hiPSC and endpoint analyses. Right panel: representative flow cytometry dot plots displaying how primitive hematopoietic cells (CD45+CD34+), mature hematopoietic cells (CD45+) and cells committed towards early neuroectoderm (A2B5+) are identified and analyzed. (**B–E**) Lines CB-hiPSC #2 and F-hiPSC #2 display a significant impairment in their differentiation capacity into primitive blood cells (CD45+CD34+) (**B**), total blood cells (CD45+CD34−) (**C**), colony-forming unit potential (**D**) and early neural progenitors (**E**). (**F**) The expression of the reprogramming factors remain upon hematopoietic (H) and neural (N) differentiation, consistent with that observed in undifferentiated hiPSC lines.

In order to assess whether hiPSC derived from CD34+ HSPCs are better able to generate hematopoietic cells than hiPSC derived from fibroblasts [51] (the so-called “epigenetic memory”) and whether the residual expression of the reprogramming factors affects hiPSC directed differentiation, we have compared side-by-side the hematopoietic and early neuroectoderm developmental potential of the four hiPSC generated: two CB-iPSC and two F-iPSC. Human iPSC were allowed to differentiate *in vitro* into blood or early neuroectoderm by EB formation as described [42], [52]. The emergence of primitive blood cells (identified as CD45+CD34+) and total blood cells (CD45+CD34−) was analyzed at day 15 and 22 of development (**Figure 3A**). The presence of hematopoietic progenitors with CFU potential was analyzed at day 15 of EB development (**Figure 3A**). Early neuroectoderm cells (identified as A2B5+) were analyzed at day 8 of EB development (**Figure 3A**).

Interestingly, as shown in **Figure 3B, C, D, E**, those hiPSC which had silenced OKSM ectopic expression (CB-iPSC#1 and F-iPSC#1) displayed fair hematopoietic (∼30% CD45+) and early neuroectoderm (∼40% A2B5+) differentiation potential. In contrast, those hiPSC which failed to switch off OKSM expression (CB-iPSC#2 and F-iPSC#2) were unable to differentiate towards either lineage (<1% CD45+ and <3% A2B5+), regardless of the source of the reprogrammed cell. The expression of the ectopic reprogramming factors remained upon hematopoietic and neural differentiation, consistent with that observed in undifferentiated hiPSC (**Figure 3F**). This indicates that the residual expression of the reprogramming factors seriously compromise hiPSC differentiation. Additionally, although no differences in the neuroectoderm differentiation potential were observed, the CB-iPSC#1 line displayed a much more efficient hematopoietic differentiation (primitive and total blood cells and CFU) than F-iPSC#1, indicative of epigenetic memory.

### Transgene-free hiPSC clones display improved differentiation capacity

**Figure 4:**
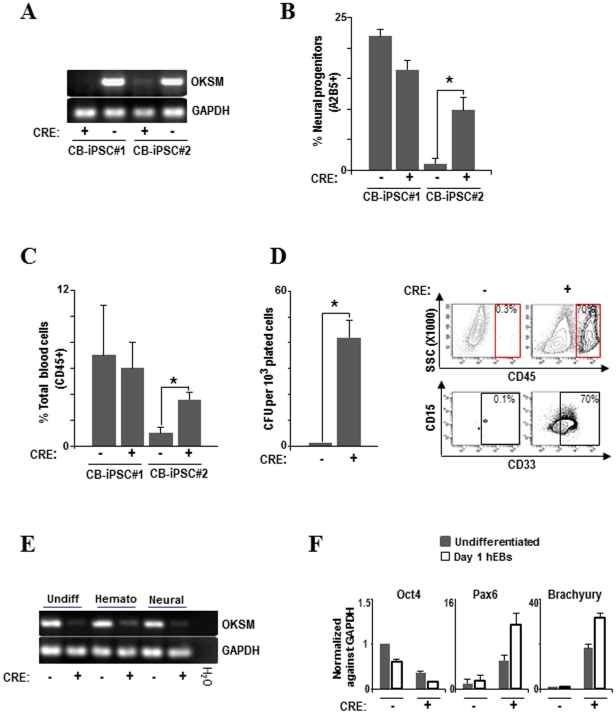
Transgene-free hiPSC clones display improved differentiation capacity. (**A**) PCR of genomic DNA (gDNA) showing successful adenovirus-based Cre-excision of the reprogramming cassette in CB-hiPSC. CB-hiPSC #2 after Cre-excision significantly improved its differentiation capacity into early neural progenitors (**B**) total blood cells (**C**) and colony-forming unit potential (**D**). Flow cytometry from CB-hiPSC #2 CFUs before and after Cre excision clearly shows that CD45+ and CD45+CD33+ myeloid cells appear in CFUs from CB-hiPSC #2 after Cre excision. (**E**) RT-PCR confirming that in CB-hiPSC #2 after Cre-excision the expression of ectopic reprogramming factors is greatly reduced in undifferentiated hiPSC and after 15 days of hematopoietic and neural differentiation. (**F**) Q-RT-PCR showing an increased expression of the early differentiation markers, PAX6 (neural) and Brachyury (mesendoderm), in CB-iPSC #2 after Cre-mediated OKSM excision. Q-RT-PCR expression is normalized against GAPDH.

To confirm that the residual expression of the reprogramming factors compromises hiPSC differentiation, we took advantage of the LoxP sites flanking the expression cassette (OKSM) to excise the OKSM transgene by infection of CB-iPSC#1 (transgene silenced) and CB-iPSC#2 (transgene not silenced) with adenoviral vectors expressing the Cre-recombinase gene under the control of the CMV promoter (Ad-CMV-Cre) [37]. Successful Cre-mediated excision of the OKSM transgene was confirmed by genomic PCR in both CB-iPSC#1 and CB-iPSC#2 (**Figure 4A**). Cre-mediated removal of OKSM did not affect either hiPSC homeostasis or the expression of pluripotency-associated surface markers and transcription factors (**Figure S2**). After Cre-mediated excision of the non-silenced OKSM transgene, the CB-iPSC#2 line significantly improved its differentiation capacity into early neural progenitors (5-fold increase; **Figure 4B**) total blood cells (4-fold increase; **Figure 4C**) and CFU progenitor potential (40-fold increase; **Figure 4D**). The abolishment of OKSM expression after Cre-mediated excision was maintained long-term upon hematopoietic and neural differentiation (**Figure 4E**). As expected, differentiation was not improved in CB-iPSC#1 infected with Ad-CMV-Cre because the OKSM transgene was already switched off in this hiPSC line (**Figure 4B,C**). In agreement with the significantly higher functional/phenotypic neural and blood differentiation, a robust increase in the expression of the early neural- and mesoderm-specific transcription factors Pax6 and Brachyury, respectively, was observed in the CB-iPSC#2 line after Cre-mediated OKSM removal (**Figure 4F**). In contrast, the expression of the pluripotency factor Oct4 dropped faster in the CB-iPSC#2 line after OKSM excision, suggesting that the residual expression of the reprogramming factors functions as a developmental brake impairing hiPSC differentiation.

## Discussion

Induced PSCs have been generated from different somatic cell types and recently also from various cell populations of CB [7], [11], [16], [17]. There is some controversy, not yet rigorously resolved in the human context, that the more immature the somatic cell to be reprogrammed, the easier and more efficient hiPSC generation is [51], [53]. If this hypothesis is confirmed, CB may represent an advantageous cell source for hiPSC generation, given that it contains more immature and cytokine-responsive subsets of HSPCs [12], [13], [51]. CB also represents a source enriched with hematopoietic, mesenchymal and endothelial progenitors. Importantly, CB cells are young newborn cells expected to accumulate minimal somatic mutations compared to other somatic tissues sourced from older subjects, and they display the immunological immaturity of neonatal tissues. Here, we report the successful derivation of fully-characterized hiPSC lines from CB-CD34+ cells. In contrast to Giorgetti *et al* [7], we generated hiPSC from CD34+ cells rather than from the CD133+ cell subset because the latter contains hematopoietic progenitor cells already committed to myeloid but not lymphoid lineages- [12], [13], [14], [15]. It is worth mentioning that Takenaka *et al* [16] succeeded very recently in the derivation of hiPSC from CB-derived CD34+ cells upon OKSM ectopic expression combined with the deletion of the p53 gene; in the presence of p53 they were unable to maintain the hiPSC longer than 1 month. Our data, however, reveals the successful generation and maintenance of hiPSC from CB-CD34+ cells harbouring wild-type alleles for p53. The differences between these two studies may be explained by the use of distinct hematopoietic cytokines, distinct viral transduction systems (retro *versus* lenti) as well as the use of different feeders (MEFs *versus* SNLs) and culture conditions.

To date, hiPSC have been generated from CB-CD34+ HSPCs using four independent retroviral vectors (pMX) expressing OKSM or upon deletion of the p53 gene. However, we succeeded in the generation of fully-characterized hiPSC from newborn CD34+ HSPCs using a single excisable polycistronic lentiviral vector based on 2A and IRES sequences expressing OKSM under the EF1α constitutive promoter, likely minimizing the number of proviral insertions. Interestingly, although it has been shown that hiPSC can be generated from cryopreserved CB, this is the first report showing successful derivation of hiPSC from fresh CB-CD34+ cells [16], [17]. Our data indicate that cryopreserved and fresh CB units compare quite well in terms of hiPSC derivation efficiency and karyotypic stability. Our hiPSC remain karyotypically stable after 30 passages and the hiPSC derivation efficiency was 0.0002% which is low but within the reported range of iPSC derivation efficiency (0.0001%–0.5%) [54], [55], suggesting that enforced transgene expression appears to initiate a sequence of stochastic events over several days that eventually induces a very small fraction of cells to acquire a stable pluripotent state. The slow reprogramming process coupled to the low reprogramming efficiency impedes detailed mechanistic studies of this technology [55], [56]. The identification of small molecules capable of enhancing the reprogramming efficiency is an active field of research. Indeed, recent studies have demonstrated that several compounds which have the capacity to modulate epigenetic-modifying enzymes exhibit positive effects on iPSC generation. For instance, treatment with the DNA methyltransferase 5-AZA and with the histone deacetylase inhibitors valproic acid, sodium butyrate or trichostatin A were capable of increasing the reprogramming efficiency [55], [56], [57].

Several studies have demonstrated that reactivation or sustained expression of reprogramming factors may result in deleterious outcomes such as tumor formation or disruption of pluripotency [58], [59], [60]. Moreover, the study of the biology of reprogramming and the evaluation of how closely iPSC and ESCs functionally resemble each other will greatly benefit from the use of homogeneous populations devoid of any residual transgene expression. We found that the ectopic expression of OKSM was fully silenced upon reprogramming in some hiPSC and was not reactivated upon differentiation, whereas other hiPSC failed to silence the transgene expression. The inability of some hiPSC to silence the reprogramming factors was associated to neither the cell type/tissue origin nor lentiviral backbone/promoters used. Integrating viral delivery methods (lenti- or retro-vectors) do insert the exogenous DNA randomly in the genome. Epigenetic remodelling and marks (i.e. promoter methylation or histone acethylation) vary throughout the genome leaving genomic slots refractory to ectopic gene expression whereas other parts of the host genome may be much less reluctant to ectopic gene expression [46], [61], [62]. It may thus be speculated that the level of epigenetic control dictated by the integration site within the host genome may explain why some iPSC clones fully silence the transgese but other iPSC clones do not.. We have compared side-by-side the hematopoietic and early neuroectoderm developmental potential of all hiPSC and we found that residual expression of the reprogramming factors functions as a developmental brake impairing hiPSC differentiation towards both hematopoietic and neuroectoderm lineages. Taking advantage of a floxed single copy of a policistronic reprogramming vector, we excised the reprogramming cassette using Ad-CMV-Cre and “transgene-free” CB-hiPSC were generated. Cre-mediated removal of OKSM did not affect either hiPSC homeostasis or the expression of pluripotency-associated surface markers and transcription factors. After Cre-mediated excision of the non-silenced OKSM transgene, CB-iPSC significantly improved the differentiation capacity into both hematopoietic cells (mesoderm) and early neural progenitors (ectoderm). Our findings emphasize the importance of vector excision prior to directed differentiation of CB-iPSC in culture if the goal is to adapt differentiation culture conditions originally developed in hESC culture systems. This is crucial not only for potential cell replacement strategies but also for drug-screening and disease modeling applications in which CB-iPSC may represent the healthy controls for hiPSC generated from a variety of primary patient leukemic blasts. Nevertheless, our findings do not prove that “transgene-free” CB-iPSC are functionally equivalent to hESCs in terms of differentiation capacity. Despite transgene-silenced and transgene-removed CB-iPSC performed well in neuroectoderm and blood-promoting culture conditions, further extensive inter-laboratory studies must be prospectively undertaken comparing the lineage-specific differentiation potential and epigenetic memory among multiple hiPSC derived from different tissues, and using distinct strategies and hESCs. In summary, we report for the first time the generation of hiPSC from fresh CB-derived CD34+ HSPCs using a single excisable polycistronic (OKSM) lentiviral vector. A direct comparison between hiPSC clones before and after Cre-mediated reprogramming excision reveals that residual expression of the reprogramming factors robustly prevents both mesoderm and neuroectoderm differentiation.

## Supporting Information

Figure S1 **hiPSC lines display** ***in vitro*** **potential for three germ layer differentiation.** Histological analysis of EBs showing spontaneous *in vitro* differentiation into ectoderm (pan-cytokeratin+), mesoderm (smooth muscle Actin+) and endoderm (neuroephitelium).
(TIF)

Figure S2 **Phenotypic and molecular characterization of CB-iPSC after Cre-mediated excision of the reprogramming transgenes.** After Cre-mediated excision of the reprogramming transgene, the CB-iPSC express the pluripotent surface markers SSEA-3, SSEA-4, Tra-1-60 and Tra-1-81 as well as nuclear Oct4 by flow cytometry (**A**) and retain expression of the pluripotency markers Oct4, Sox2, Nanog and Rex1 by RT-PCR (**B**).
(TIF)
