# Supplementary figures and images for: Residual Expression of the Reprogramming Factors Prevents Differentiation of iPSC Generated from Human Fibroblasts and Cord Blood CD34+ Progenitors
Source: PLoS One. 2012 Apr 24;7(4):e35824. doi: 10.1371/journal.pone.0035824 (PMC3335819; doi:10.1371/journal.pone.0035824)

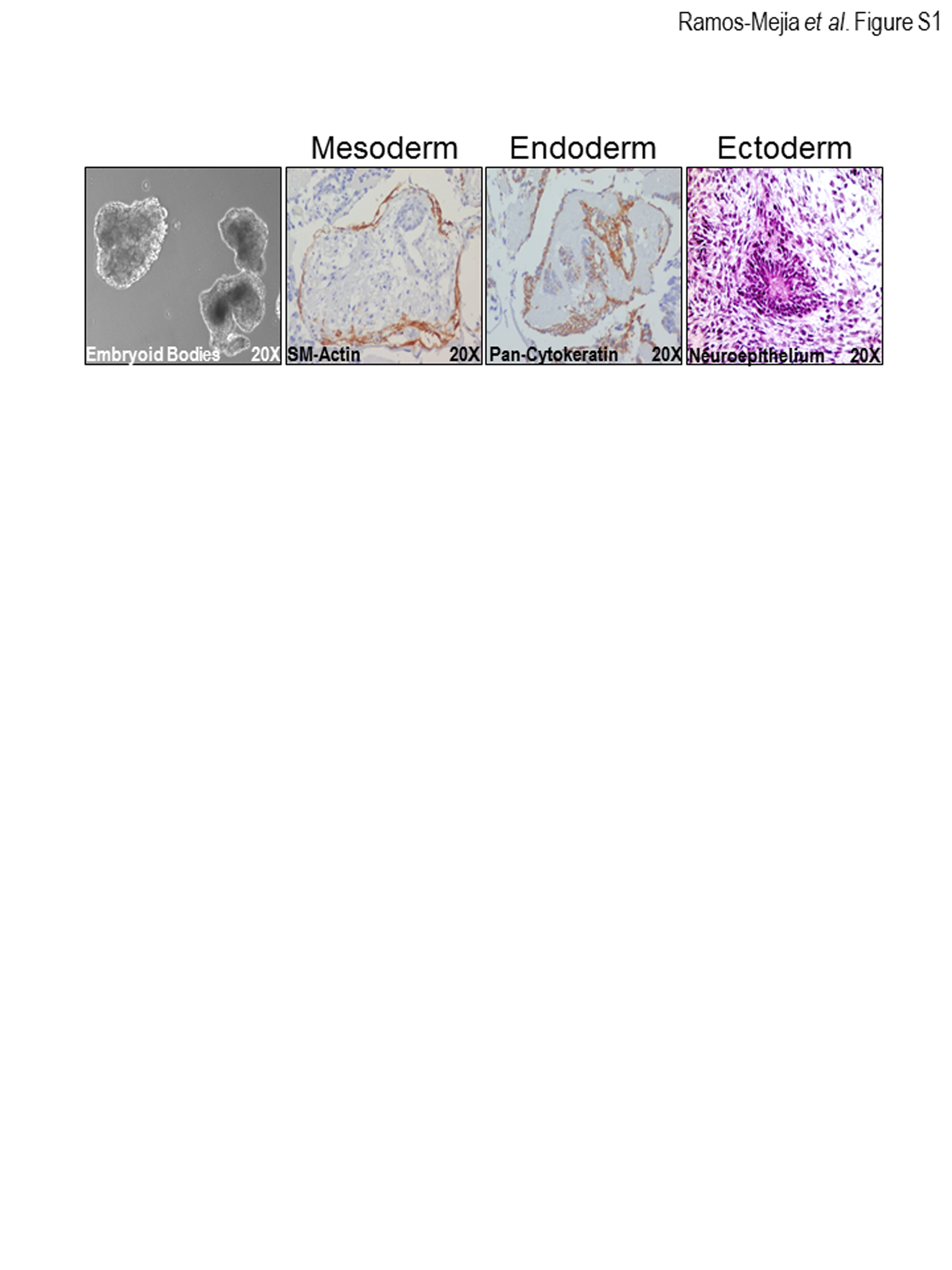

Supplement: Figure S1 — hiPSC lines display in vitro potential for three germ layer differentiation. Histological analysis of EBs showing spontaneous in vitro differentiation into ectoderm (pan-cytokeratin+), mesoderm (smooth muscle Actin+) and endoderm (neuroephitelium). (TIF) [file pone.0035824.s001.tif]

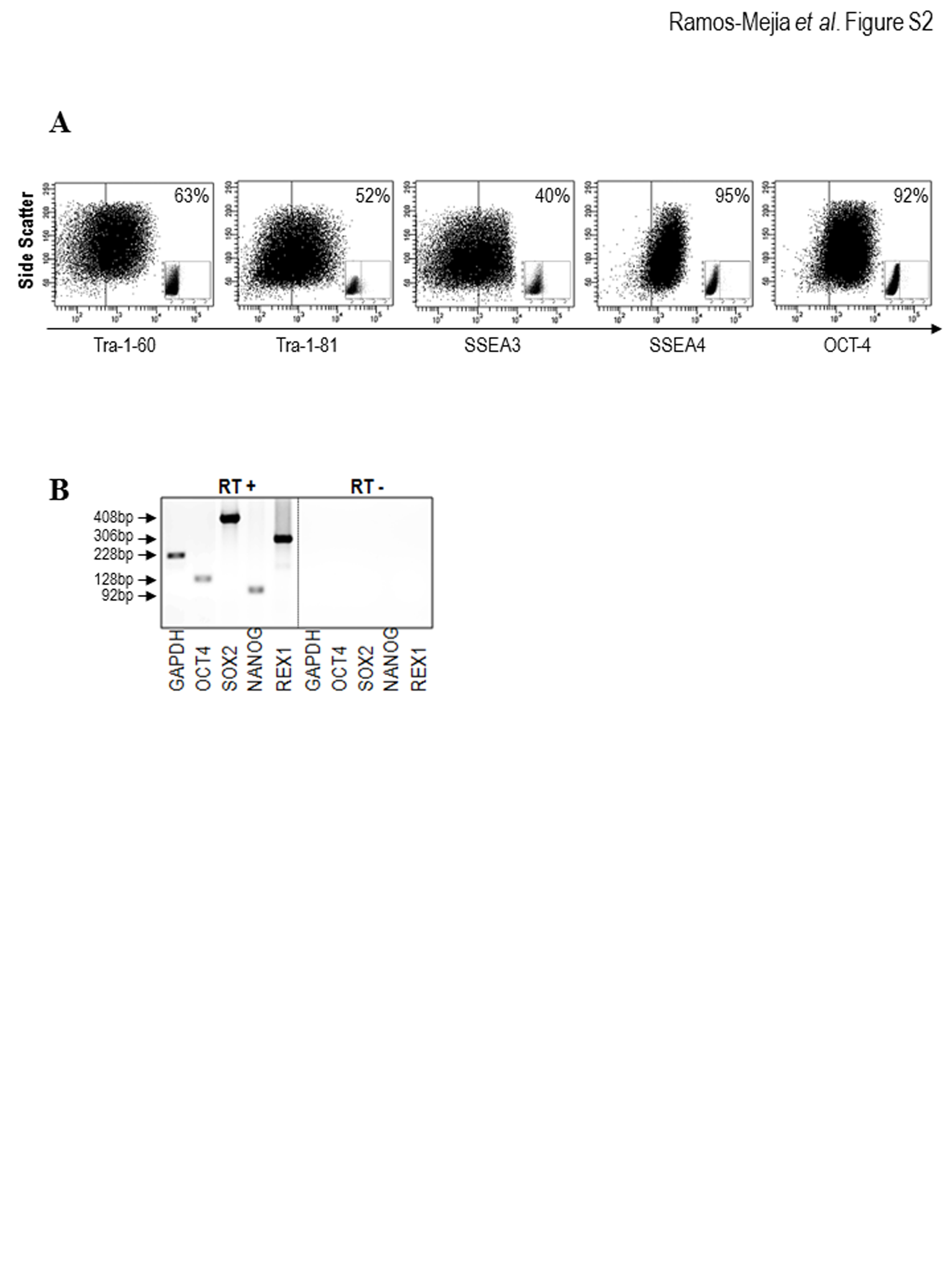

Supplement: Figure S2 — Phenotypic and molecular characterization of CB-iPSC after Cre-mediated excision of the reprogramming transgenes. After Cre-mediated excision of the reprogramming transgene, the CB-iPSC express the pluripotent surface markers SSEA-3, SSEA-4, Tra-1-60 and Tra-1-81 as well as nuclear Oct4 by flow cytometry (A) and retain expression of the pluripotency markers Oct4, Sox2, Nanog and Rex1 by RT-PCR (B). (TIF) [file pone.0035824.s002.tif]
